# Analysis of Antioxidant Activity and Flavonoids Metabolites in Peel and Flesh of Red-Fleshed Apple Varieties

**DOI:** 10.3390/molecules25081968

**Published:** 2020-04-23

**Authors:** Xiang Zhang, Jihua Xu, Zhaobo Xu, Xiaohong Sun, Jun Zhu, Yugang Zhang

**Affiliations:** 1 Qingdao Key Laboratory of Genetic Development and Breeding in Horticultural Plants, Qingdao Agricultural University, Qingdao 266109, China; 18306391375@163.com (X.Z.); xujihua@qau.edu.cn (J.X.); mingsun9887@163.com (X.S.); junzhu@qau.edu.cn (J.Z.); 2 College of Horticulture, Qingdao Agricultural University, Qingdao 266109, China; 3 College of Life Sciences, Qingdao Agricultural University, Qingdao 266109, China; 4 Qingdao Agriculture Technology Extension Center, Qingdao 266071, China; 18661683886@163.com

**Keywords:** red-fleshed apple, flavonoids, anthocyanins, polyphenols, antioxidant activity, metabolites

## Abstract

In this research, we compared the phenotypical characters, total anthocyanins content, total phenols content, and antioxidant activity of red-fleshed apple cultivars ‘XJ4’, ‘QN-5’, ‘DH’ and ‘HX1’ at three fruit developmental stages. A further flavonoids metabolites study was conducted in ‘XJ4’ and ‘DH’. We found broader variation of total anthocyanins content in the peel of the four cultivars, which might result in larger differences of free radicals scavenging rate. The most significant difference in fruit phenotype, anthocyanins content, and DPPH scavenging rate was observed between ‘XJ4’ and ‘DH’ at mature stage. Therefore, the flavonoids metabolites of ‘XJ4’ and ‘DH’ at mature stage were compared to unveil the details of anthocyanins compounds. The unique compounds pelargonidin 3-*O*-β-d-glucoside and cyanidin-3-*O*-malonylhexoside were detected only in peel and flesh of ‘XJ4’ but not in ‘DH’, which might contribute to the purple peel and dark-red flesh color of ‘XJ4’. Significantly decreased upstream metabolites in the early biosynthetic genes regulated domain were found only in ‘XJ4’ peel but not in the flesh. This might explain why the anthocyanins content in ‘XJ4’ peel was decreased largely at the mature stage. Taken together, our findings will give some insight into the metabolites study in flavonoid biosynthetic pathway of red-fleshed apple.

## 1. Introduction

Flavonoids are a big class of polyphenolic plant secondary metabolites, including flavonols, flavones, isoflavones, anthocyanins and so on [1]. Most of the flavonoids are effective natural antioxidants [2]. In human beings, flavonoids play important roles in maintaining normal vascular permeability and protecting against diseases such as hyperglycemia, cancer, and diabetes [3]. Anthocyanins are one group of the major secondary metabolites that cause plants to exhibit different colors. Fruits, as a group of important horticultural products, contain a wide variety of colors such as red, pink, purple and blue contributed by anthocyanins [4]. The six most commom anthocyanidin pigments in fruits are cyanidin (30%), delphinidin (22%), pelargonidin (18%), peonidin (7.5%), malvidin (7.5%) and petunidin (5%) [5,6]. The anthocyanidin structure contains two aromatic benzene rings and an oxygenated heterocycle with three carbons, the hydroxyl group at C3 or C5 in the anthocyanidin molecule can be connected with glucose, rhamnose, galactose or some pentose to form anthocyanins [7,8]. The number of hydroxyl groups on two aromatic benzene rings and the double bond positions on oxygenated heterocycle have a great impact on the sensitivity of anthocyanins to oxidation [9].

As strong antioxidants, anthocyanins have a strong DPPH, ·OH, and O_2_·- radicals scavenging ability and the related studies have been intensively reported in many horticultural crops [10]. Previous study found the extract of anthocyanins from purple cabbage had strong capacity to scavenge free radicals [11]. Lyophilized pomegranate anthocyanins extract had strong ability to scavenge hydroxyl and superoxide anions [12]. Purple cauliflower had high total anthocyanins contents and scavenging rate of DPPH radical [13]. Red-fleshed apples (*Malussieversii f. Neidzwetzkyana* (Dieck) Langenf) are valuable resources attracting much more attention because they contain extremely high levels of anthocyanins compared to white and off-white fleshed apples [14]. The enriched anthocyanins properties of red-fleshed apple made it show stronger antioxidant activity. Our previous study indicated red-fleshed apple cultivar ‘QN-5’ exhibited stronger capacity for DPPH, OH, and O_2_·- radicals scavenging when compared to vitamin C [15]. A recent study found red-fleshed apples can inhibit the proliferation of human breast cancer MCF-7 and MDA-MB-231 cells through studying the antioxidant and anti-proliferation properties, compared with a traditional cultivar ‘Fuji’ [16]. In addition, it’s reported anthocyanins extract from red-fleshed apple could eliminate ROS induced oxidative damage in porcine cells [17]. Interestly, it has been found that the antioxidant activity and phenolic content in peel of red-fleshed apple were higher than that in the flesh and whole apple [18].

Metabolomics is the quantitative study of the metabolite components of integrated living systems, reflecting both endogenous (physiological and developmental) and exogenous (environmental) dynamic changes [19]. Previously, the techniques for detecting metabolites were mainly depended on single technique such as column chromatography, gas chromatography (GC) and liquid chromatography (LC), then the integrated approaches like gas chromatograph-mass spectrometer (GC-MS) and liquid chromatograph-mass spectrometer (LC-MS) became promising tools for the study of plant metabolites diversity [20,21]. Recently, more advanced technique LC-Electrospray Ionization−tandem mass spectrometry (LC-ESI-MS/MS) has emerged and been widely used in flavonoids study [22,23,24]. Nowadays, the relationship between differential metabolites profiles and biochemical properties could be analyzed based on the advanced metabolomics techniques [24]. The cutting edges of metabolomics approaches allow more accurate and precise identification of metabolites in biosynthetic pathway. Significant different metabolites in flavonoid biosynthetic pathway were reported in purple fig mutation compared to the green control [23]. The interaction of the primary and secondary metabolites profiles combined with proteomics elucidated the accumulation of phenolic substances and other major compounds related to flavonoids accumulation during seed development in cacao [25].

Over the past decade, studies related to red-fleshed apple were mainly focused on the structural and regulatory genes which play important roles in anthocyanins biosynthesis [26,27,28,29]. Little attention was paid to the identification and classification of metabolites of red-fleshed apple. Therefore, in this study we carried out antioxidant activity and metabolomics analysis using red-fleshed apple as material. Before the metabolites study, we comprehensively investigated the total anthocyanins content, total phenols content, and antioxidant activity using four phenotypically different red-fleshed apple cultivars ‘XJ4’, ‘QN-5’, ‘DH’ and ‘HX1’ at three different stages. We found ‘XJ4’ and ‘DH’ showed the biggest difference in phenotypical and physiological traits detected. The compounds pelargonidin 3-*O*-β-d-glucoside and cyanidin-3-*O*-malonylhexoside significantly increased in ‘XJ4’ but not in ‘DH’, which might contribute to the purple peel and dark-red flesh of ‘XJ4’.

## 2. Results and Discussion

### 2.1. Phenotypic Characters

**Figure 1:**
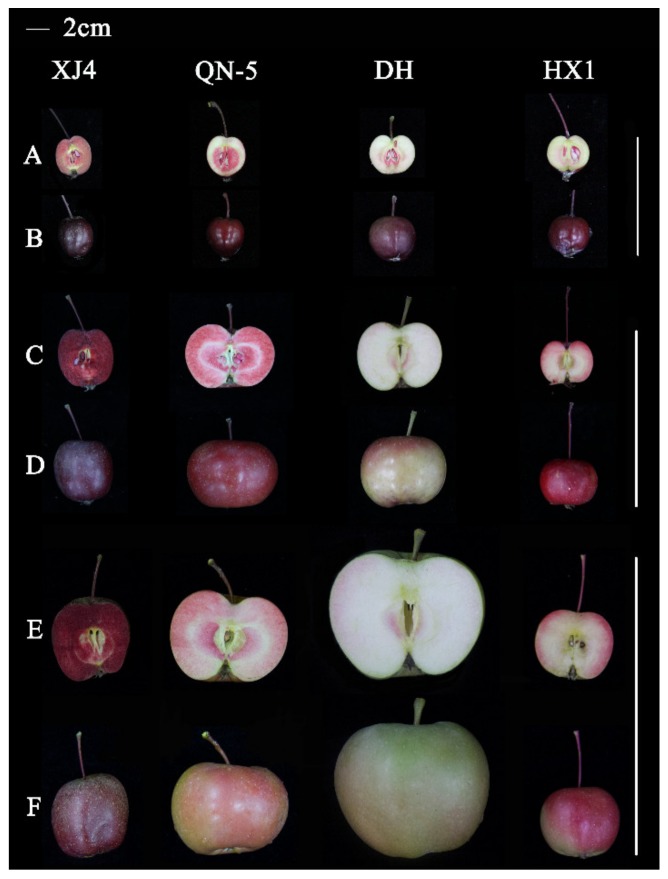
Fruit phenotypic characters of four different red-fleshed apple cultivars at young, developmental and mature stages. The longitudinal section and intact fruit at young stage (**A**,**B**), developmental stage (**C**,**D**), and mature stage (**E**,**F**) were present.

At young stage, the flesh color of ‘XJ4’ and ‘QN-5’ was red while only very light red flesh was observed in ‘DH’ and ‘HX1’ (Figure 1A). There was no obvious difference for peel color and fruit size among the four red-fleshed apple cultivars (Figure 1B). However, with the development of fruit, significant difference of both peel and flesh color, and fruit shape began to appear (Figure 1C,D). The darkest red color, nearly purple red, of peel and flesh was observed in ‘XJ4’, followed by ‘QN-5’. On the contrary, ‘DH’ had the lightest peel and flesh color. At the mature stage, ‘XJ4’ still had dark red peel and flesh, but the red peel and flesh of ‘QN-5’ was partially faded away, which might be caused by the increased fruit size (Figure 1E,F). The ‘DH’ showed the largest fruit size. The most significant difference was observed between ‘XJ4’ and ‘DH’ peel and flesh color, and fruit size at mature stage. According to the previous report, red-fleshed apple can be classified into two types [30,31]. The characteristics of type I red-fleshed apple is red pigment in fruit core and cortex, while type II red-fleshed apple has white core, only fruit cortex accumulate red pigments. Here, all of the four red-fleshed apple cultivars exhibited the characters of type I category.

### 2.2. Total Anthocyanins and Total Phenols Content 

The anthocyanins content in both peel and flesh of red-fleshed apple cultivars ‘XJ4’, ‘QN-5’, and ‘DH’ had decreased gradually with the development of fruit, but the anthocyanins content of ‘HX1’ remained similar level (Table 1). When compared the anthocyanins content in peel and flesh, the overall tendency was that anthocyanins in peel was higher than flesh, except for ‘DH’ (developmental and mature stage) and ‘QN-5’ (developmental stage). ‘XJ4’ had the largest level of anthocyanins content in both peel and flesh at all three stages, while ‘DH’ showed the lowest level. At young fruit stage, total anthocyanins content in peel of ‘XJ4’ was 1702.6 mg·kg^−1^ FW, followed by ‘QN-5’, whereas the peel of ‘HX1’ (109.1 mg·kg^−1^ FW) and ‘DH’ (170.6 mg·kg^−1^ FW) showed the lowest amount. In flesh, the anthocyanins content of ‘XJ4’ was approximately one third of the amount in peel. At developmental stage, total anthocyanins content in peel and flesh of ‘XJ4’ and ‘HX1’ was similar to the levels of young stage. The peel of ‘QN-5’ and ‘DH’ anthocyanins content was significantly lower than the content of young stage. At mature fruit stage, total anthocyanins content in peel of ‘XJ4’ was 441.0 mg·kg^−1^ FW, which was similar to the content in flesh of ‘XJ4’. The average value of anthocyanins content in peel and flesh of ‘XJ4’ at mature stage was comparable to our previous report of anthocyanins content in the mixture extract of peel and flesh of ‘XJ4’ [32]. The lowest amount of anthocyanins in peel and flesh was observed in ‘DH’, with 12.1 mg·kg^−1^ FW and 24.3 mg·kg^−1^ FW respectively.

The differences of total phenols content in both peel and flesh among the four apple cultivars was not as significant as total anthocyanins content. We found the total phenols content in peel was always higher than that in flesh at all three stages, which confirmed the results in previous study using 12 apple cultivars to compare the phenolic compounds contents in peel and flesh [33]. Total phenols content in peel of ‘XJ4’ and ‘HX1’ at young stage was the highest, with 8114.4 and 9082.3 mg·kg^−1^ FW, respectively, whereas the flesh of ‘QN-5’ at young stage showed the lowest amount (3233.6 mg·kg^−1^ FW). In addition, ‘HX1’ also contained the highest content of total phenols in flesh, with 5346.1 mg·kg^−1^ FW. At the developmental stage, total phenols content in peel of ‘XJ4’ was the significantly higher than others, whereas the flesh of ‘DH’ and ‘QN-5’ showed the lowest amount. Total phenols content in both peel and flesh of ‘XJ4’ and ‘HX1’ at mature stage was significantly higher than the other two cultivars. ‘QN-5’ peel and flesh showed the lowest amount, only 1239.6 and 327.6 mg·kg^−1^ FW respectively. Among the four red-fleshed apple cultivars, the anthocyanins differences between ‘XJ4’ and ‘DH’ were the most significant.

### 2.3. Antioxidant Activity of Anthocyanins Extract from Peels and Flesh 

In order to fairly compare the antioxidant activity of anthocyanins extract in four phenotypically different red-fleshed apples, all of the anthocyanins extract were diluted or concentrated as needed to reach the same concentration (50 mg·kg^−1^ FW). The dilution ratio of ‘XJ4’ was the highest due to high anthocyanins content, while the dilution ratio of ‘DH’ was the lowest. The anthocyanins extract of all four different red-fleshed apples had a stronger ability to scavenge DPPH radical, ·OH, and O_2_·- when compared with vitamin C (VC) at all three stages. Further, among the three types of free radicals, higher ability to scavenge DPPH radical was detected than to remove ·OH, and O_2_·- (Table 2). At young stage the lowest anthocyanins extract scavenging rate on DPPH radical and ·OH was from peel of ‘XJ4’ with the levels of 57.7% and 14.3% respectively. At developmental stage, the anthocyanins extract from peel of ‘XJ4’ and ‘QN-5’ showed significantly higher level scavenging rates of DPPH radical (90.7% and 94.7% respectively). The scavenging rate of anthocyanins extract from peel of ‘DH’ on DPPH radical was the lowest with the percentage of 48.5%. For ·OH scavenging rate, the highest (54.0%) was anthocyanins extract from flesh of ‘QN-5’ and the lowest (13.8%) was from flesh of ‘DH’. Similar to the results of scavenging rate of DPPH, ‘XJ4’ and ‘QN-5’ peel also exhibited higher level of O_2_·- scavenging rate than other two red-fleshed apple cultivars. At mature stage, the scavenging rate of anthocyanins extract from peel and flesh of ‘DH’ on DPPH radical was 21.4% and 46.5% respectively, which was significantly lower than other three cultivars. ‘XJ4’ flesh anthocyanins extract showed significantly high ·OH scavenging rate, with the level of 75.1%. In addition, there was no significant difference in the scavenging rate of O_2_·- by anthocyanins extract from peel and flesh of all red-fleshed apple. 

Through the study of antioxidant activity of anthocyanins extract, we found that generally the DPPH radical scavenging rate was positively related to anthocyanins content. Recent study in apple also reported higher total phenolic compounds was related to stronger antioxidant activity [33]. In addition, at young stage the anthocyanins extract from peel of ‘XJ4’ showed the lowest level of DPPH radical scavenging, which was due to the higher dilution ratio (34.0) of the ‘XJ4’ extract. At the later two stages, although with higher dilution ratio (30.1 and 8.8 respectively) the DPPH radical scavenging rate of ‘XJ4’ and ‘QN-5’ were significantly higher than the others. When compare the antioxidant activity of anthocyanins extract from peel and flesh, we found that the differences of DPPH radical scavenging rate tended to be larger in the peel than in the flesh among the four red-fleshed apple cultivars examined. This might be associated with the broader variation of total anthocyanins content in the peel than the flesh of the four red-fleshed apple cultivars.

Taken together, the most significant differences for fruit phenotype, anthocyanins content, and antioxidant activity (mainly DPPH) was observed between ‘XJ4’ and ‘DH’ at mature stage. Therefore, ‘XJ4’ and ‘DH’ at mature stage were used as materials for metabolomics analysis.

### 2.4. Distribution and Cluster Analysis of Significantly Differential Metabolites in ‘DH’ vs. ‘XJ4’

**Figure 2:**
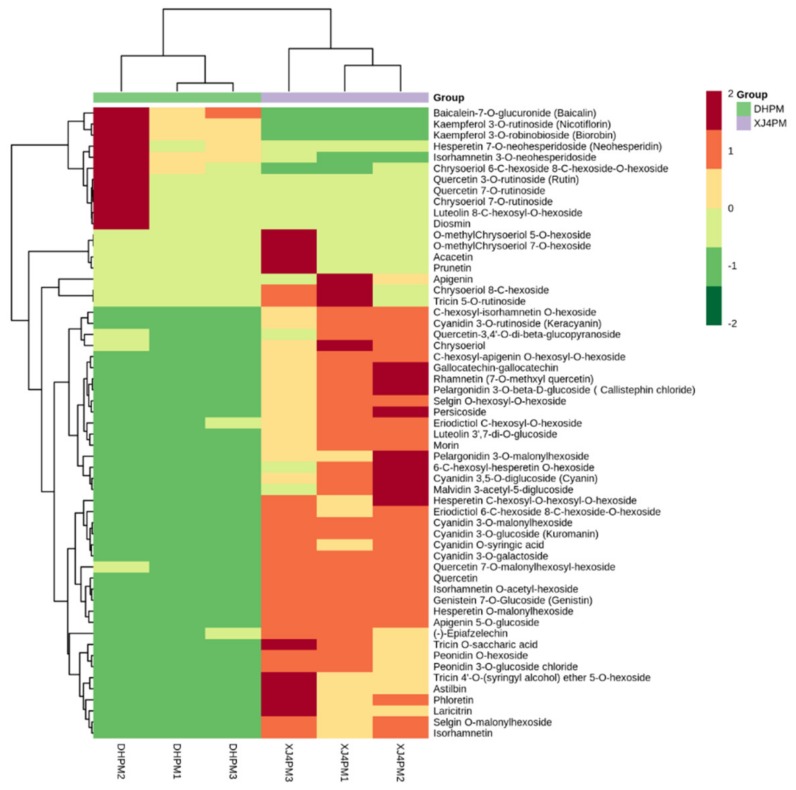
Heat map of significantly differential flavonoids metabolites in DHPM vs. XJ4PM. Red and green color indicates the content of significant differential metabolites, respectively. Columns and rows represent samples and individual metabolites, respectively. The depth of color indicates the value of correlation coefficient.

**Figure 3:**
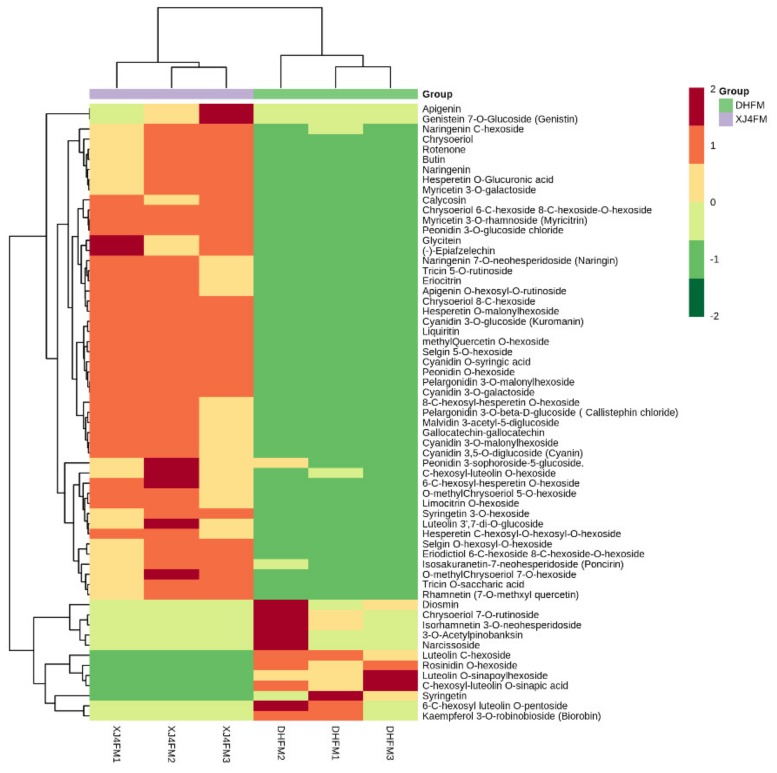
Heat map of significantly differential flavonoids metabolites in DHFM vs. XJ4FM. Red and green color indicates the content of significant differential metabolites, respectively. Columns and rows represent samples and individual metabolites, respectively. The depth of color indicates the value of correlation coefficient.

The metabolites were analyzed qualitatively and quantitatively by QC sample mass spectrometry and MRM detection to ensure the accuracy and the repeatability of data. Furthermore, PCA score plots, OPLS-DA score plots and permutation test proved that the metabolomics model was stable and reliable (Figure S1). A total of 191 differential metabolites were detected in ‘DH’ peel (DHPM), ‘DH’ flesh (DHFM), ‘XJ4’ peel (XJ4PM) and ‘XJ4’ flesh (XJ4FM). The normalized and transformed data of metabolites and samples in peel and flesh was presented by two clusters of heat maps (Figure 2 and Figure 3). For peel comparison, a clear separation of the metabolites between ‘XJ4’ and ‘DH’ was observed in all three replicates studied. Compared to DHPM, the XJ4PM exhibited significant upregulation of the flavonoids metabolites, such as cyanidin 3-*O*-malonylhexoside, cyanidin 3-*O*-glucoside (kuromanin), and cyanidin 3-galactoside (Figure 2).

**Figure 4:**
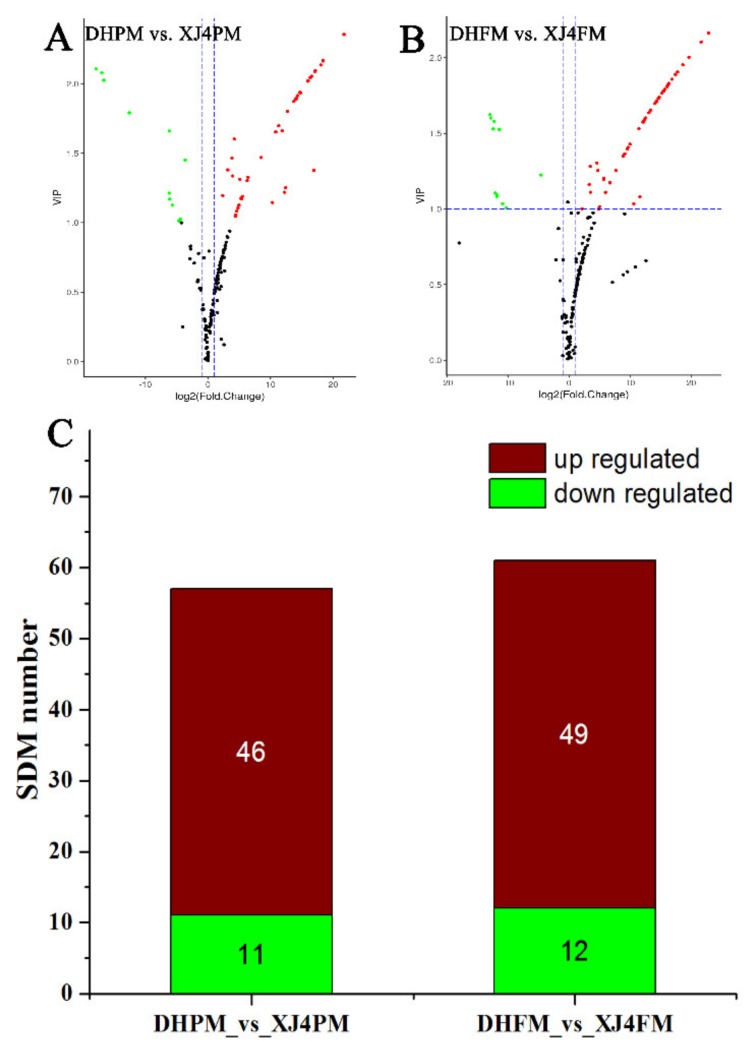
Volcano plot and number of upregulated and downregulated significantly differential flavonoids metabolites in four comparison groups. (**A**,**B**) Volcano plot of DHPM vs. XJ4PM and DHFM vs. XJ4FM. Green, red and black dots represents the number of significantly downregulated, upregulated and unchanged metabolites. (**C**,**D**) Number of significantly upregulated and downregulated metabolites (SDM) in two comparison groups DHPM vs. XJ4PM and DHFM vs. XJ4FM. SDM represents significantly differential metabolites.

Further analysis indicated there were totally 46 significantly upregulated metabolites in XJ4PM when compared to DHPM, while there were 11 significantly downregulated metabolites (Figure 4A,C). In the comparison between DHFM and XJ4FM, similarly the XJ4FM showed significant upregulation of the flavonoids metabolites, such as cyanidin 3-*O*-malonylhexoside, cyanidin 3-*O*-glucoside, cyanidin 3-galactoside, and liquiritin (Figure 3).

**Figure 5:**
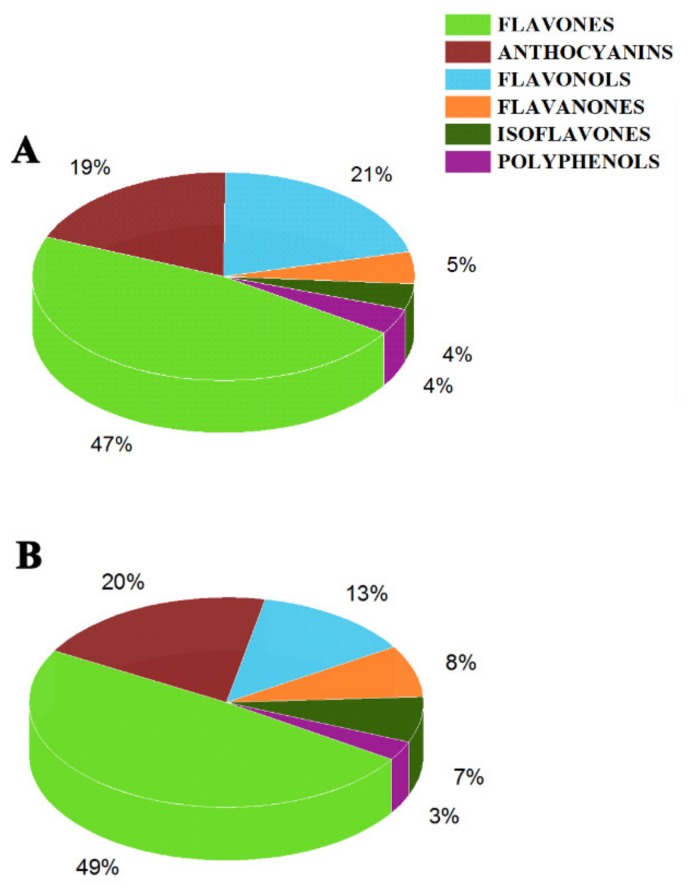
The proportion of significantly differential flavonoids metabolites in peel and flesh comparison groups. (**A**) DHPM vs. XJ4PM, (**B**) DHFM vs. XJ4FM.

In total 49 significantly upregulated metabolites were found in XJ4FM, while there were 12 significantly downregulated metabolites (Figure 4B,C). Significantly differential flavonoids metabolites were further classified into six categories including flavones, anthocyanins, flavonols, flavanones, isoflavones and polyphenols (Figure 5). Flavones accounted for the highest proportion, followed by anthocyanins and flavonols. These three largest categories make up to 87% and 82% of all the significantly differential metabolites in the two groups (Figure 5). The results indicated the flavones, anthocyanins, and flavonols are the main metabolites in the peel and flesh of ‘DH’ and ‘XJ4’. In addition, the biggest difference between peel and flesh lied in flavonols group, with the proportion of 21% and 13% in peel and flesh respectively. 

### 2.5. Significantly Differential Metabolites Assay in DHPM vs. XJ4PM and DHFM vs. XJ4FM

All of the significantly differential flavonoids metabolites profiles of peel and flesh from ‘XJ4’ and ‘DH’ fruits were presented in Table S1. Here we defined the metabolites as undetected when the content value was lower than 9.00E + 00. The significant differences of metabolites were set as variable importance in projection (VIP) ≥ 1 and fold change ≥ 2 (upregulation) or ≤ 0.5 (downregulation). The extremely significantly different metabolites were defined as the metabolites that were present in ‘XJ4’ and absent in ‘DH’ or present in ‘DH’ and absent in ‘XJ4’ (Table 3).

#### 2.5.1. Anthocyanins

The individual components and levels of anthocyanins, as a group of important flavonoid compounds, determine the different colors of apple [34,35]. In the peel group, there were three types of anthocyanidins components detected only in ‘XJ4’, namely pelargonidin, cyanidin, and malvidin. The content of pelargonidin 3-*O*-β-d-glucoside (callistephin chloride) was the largest, with 3,490,000-fold increase in XJ4PM compared to DHPM, whereas pelargonidin 3-*O*-malonylhexoside was the lowest, with 1820-fold high in XJ4PM (Table 3). The second most abundant anthocyanin was cyanidin-3-*O*-malonylhexoside with 147,000-fold increase in XJ4PM.

The flesh group contained more diverse anthocyanidins, in addition to the three types of anthocyanidins observed in peel, there were peonidin and rosinidin. The same as in peel, the content of pelargonidin 3-*O*-β-d-glucoside was the most, with 7,540,000-fold high in XJ4FM compared to DHFM, which was over twice of the level in peel. The second most abundant anthocyanidin in XJ4FM was still cyanidin-3-*O*-malonylhexoside. The peonidin *O-*hexoside and peonidin 3-*O*-glucoside chloride were found with 83,300- and 79,100-fold increase in XJ4FM. Interestingly, the rosinidin *O-*hexoside was only exist in ‘DH’ flesh but not in ‘DH’ peel, which might contribute to the higher total anthocyanins content and DPPH scavenging rate in ‘DH’ flesh than ‘DH’ peel.

A previous study has shown that cyanidin 3-*O*-galactoside was the main factor contributing to the red color in type I red-fleshed apple [36]. However, cyanidin 3-*O*-galactoside was not observed with extreme significance in both XJ4PM and XJ4FM when compared to DHPM and DHFM in our research. We also found pelargonidin 3-*O*-β-d-glucoside and cyanidin-3-*O*-malonylhexoside were the unique compounds in both ‘XJ4’ peel and flesh. The reasons why these two types of anthocyanins components levels were the largest are not clear. The findings that pelargonidin-based anthocyanins were the dominant component in peel and flesh of ‘XJ4’ was consistent with the research in red potatoes [28,37]. What’s more, in our recent publication we reported the most abundant anthocyanins accumulation was cyanidin-3-*O*-malonylhexoside in ‘XJ4’ when compared to another red-fleshed cultivar ‘RL’ in the mixed peel and flesh extraction [32].

#### 2.5.2. Flavones

For the flavones in the peel, selgin *O-*hexosyl-*O*-hexoside, tricin *O-*saccharic acid, 6-C-hexosyl-hesperetin *O-*hexoside, and luteolin 3′,7-di-*O*-glucoside were the four components with the highest contents in the XJ4PM. Baicalein-7-*O*-glucuronide (baicalin) and chrysoeriol 7-*O*-rutinoside had extremely significantly high contents only in the DHPM. In the flesh comprision, 6-C-hexosyl-hesperetin *O-*hexosideluteolin 3′,7-di-*O*-glucoside, and selgin *O-*hexosyl-*O*-hexoside were the three largest metabolites in XJ4FM. Chrysoeriol 7-*O*-rutinoside, C-hexosyl-luteolin *O-*sinapic acid, luteolin C-hexoside, and so on were only detected extremely significantly high in the DHFM.

#### 2.5.3. Flavonols, Flavanones, Isoflavones and Polyphenols

The contents of quercetin and laricitrin were extremely higher in XJ4PM while kaempferol 3-*O*-rutinoside (nocotiflorin) was the specific metabolite of DHPM. In the flesh, isorhamnetin 3-*O*-neohesperidoside, kaempferol 3-*O*-robinobioside (biorobin), and syringetin demonstrated extremely significantly high contents in the DHFM. For the flavanones, only hesperetin *O-*malonylhexoside and naringenin were detected 13,500- and 21,300-fold in ‘XJ4’ peel and flesh compared to ‘DH’. In the XJ4PM, genistein 7-*O*-glucoside (genistin) was found with 20,800-fold increment. The content of gallocatechin-gallocatechin was 3,230,000-fold higher in XJ4FM compared to DHFM.

### 2.6. KEGG Enrichment Analysis of Significantly Differential Metabolites

**Figure 6:**
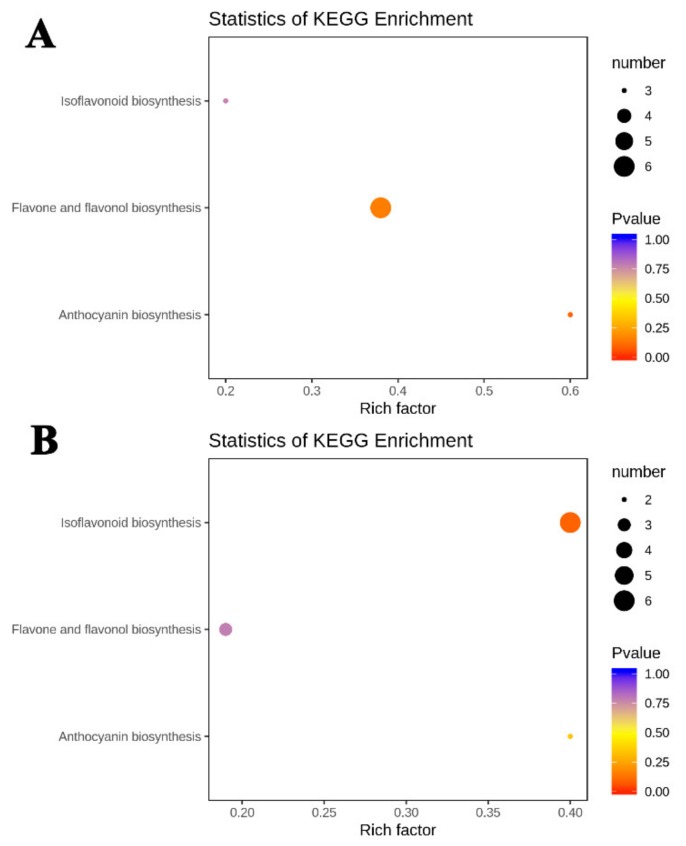
Enrichment analysis of KEGG pathway. (**A**,**B**) present the enrichment analysis of KEGG pathway in DHPM vs. XJ4PM, DHFM vs. XJ4FM respectively. The color and size of the dots represented p value and the amount of enriched differential metabolites, respectively. Rich factor means the ratio of the number of differential metabolites to the total number of metabolites enriched in a specific category.

In order to better understand which categories the differential metabolites fell into, the metabolites were mapped to KEGG metabolic pathways. KEGG enrichment analysis suggested that the significantly differential metabolites were mainly involved in three biosynthesis pathways, namely isoflavonoid biosynthesis pathway, flavone and flavonol biosynthesis pathway, and anthocyanin biosynthesis pathway (Figure 6). The largest group of differential metabolites in DHPM vs. XJ4PM was in flavone and flavonol biosynthesis category, with the number of five (Figure 6A). The largest rich factor was found in anthocyanin biosynthesis pathway, which was 0.6 in DHPM vs. XJ4PM, followed by flavone and flavonol biosynthesis pathway. By contrast, in the DHFM vs. XJ4FM, the largest group of differential metabolites was in isoflavonoid biosynthesis pathway (Figure 6B). The rich factors of isoflavonoid biosynthesis pathway and anthocyanin biosynthesis pathway were both 0.4 with the greatest enrichment degree. Therefore, significantly differential metabolites in peel and flesh comparison groups were mainly distributed in isoflavonoid biosynthesis pathway and anthocyanin biosynthesis pathway. 

### 2.7. Profiles of Differential Metabolites in Flavonoid Biosynthetic Pathways

**Figure 7:**
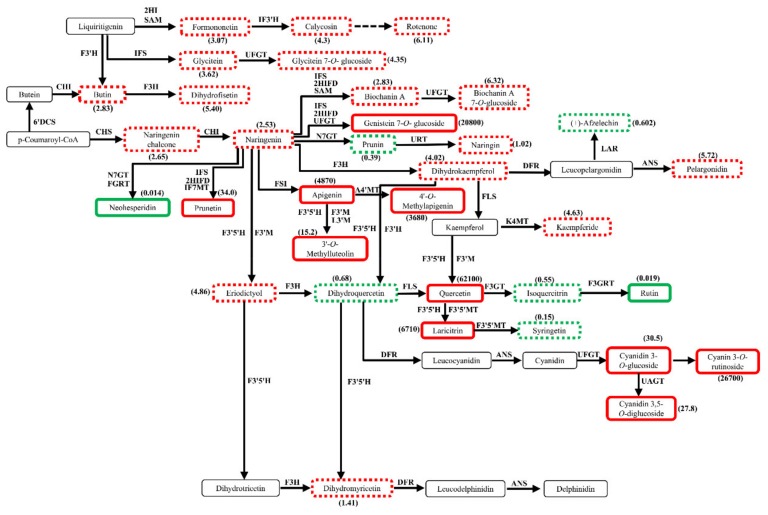
Profiles of differential metabolites in flavonoid biosynthesis pathways in DHPM vs. XJ4PM. The box in the pathway represents differential metabolites. Red and green represent upregulated and downregulated metabolites in content. Solid lines and dotted lines represent significant differences and insignificant differences, respectively.

The annotated differential metabolites in flavonoid biosynthetic pathway in DHPM vs. XJ4PM were presented in Figure 7. Due to low signal or lack of information in the database, not all of the differential metabolites were annotated by KEGG in the corresponding pathway. The naringenin is an important compound as it is the precursor for flavones, isoflavones, flavonols and anthocyanins [38]. Dihydrokaempferol, dihydroquercetin, and dihydromyricetin, which were three dihydroflavonols for the anthocyanins components pelargonidin, cyanidin, and delphinidin, were all derived from naringenin by different enzyme catalysis. We found there was no significant difference for naringenin in the peel of ‘DH’ and ‘XJ4’. However, the downstream flavones and flavonols compounds, prunetin, apigenin, 4′-*O*-methylapigenin, 3′-*O*-methylluteolin, and isoflavone genistein 7-*O*-glucoside were all significantly increased in the peel of ‘XJ4’ compared to ‘DH’, especially genistein 7-*O*-glucoside (20,800-fold). The downstream compounds quercetin and laricitrin are the main flavonol metabolites, which were also significantly increased in the peel of ‘XJ4’ compared to ‘DH’. Interestingly, the anthocyanins components cyanidin 3-*O*-glucoside, cyanin 3-*O*-rutinoside, and cyanidin 3,5-*O*-diglucoside were significantly increased in ‘XJ4’ peel compared to ‘DH’. On the contrary, only two metabolites, neohesperidin and rutin, were detected significantly increased in ‘DH’ peel. Based on the previous reports, two types of genes, regulatory genes and structural genes, are involved in anthocyanins biosynthetic pathway, and structural genes can be further classified into early biosynthetic genes and late biosynthetic genes [28,39]. The genes located upstream of chalcone isomerase (CHI) and including CHI are early biosynthetic genes, with the downstream genes as late biosynthetic genes. CHI stereospecifically catalyzes the intramolecular cyclization of naringenin chalcone to produce naringenin. Interestingly, in our research, we found the significantly increased metabolites consistently existed in the late biosynthetic genes regulated domain in ‘XJ4’ peel when compared to ‘DH’ peel. The metabolites regulated by the early biosynthetic genes, including naringenin, did not show significant difference in the peel of ‘DH’ and ‘XJ4’.

**Figure 8:**
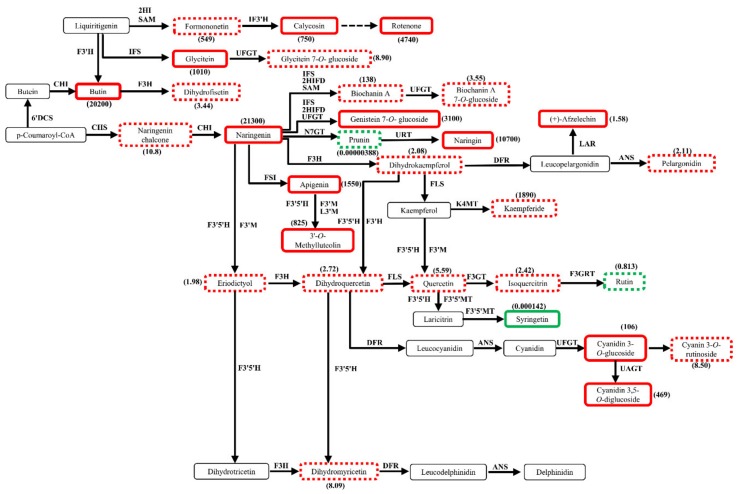
Profiles of differential metabolites in flavonoid biosynthetic pathways in DHFM vs. XJ4FM. The box in the pathway represents differential metabolites. Red and green represent upregulated and downregulated metabolites in content. Solid lines and dotted lines represent significant differences and insignificant differences, respectively.

The detected differential metabolites in flavonoid biosynthetic pathway in DHFM vs. XJ4FM were different from DHPM vs. XJ4PM (Figure 8). Naringenin, which was not detected in the differential metabolites analysis in peel study, here it had significantly higher levels (21,300-fold) in ‘XJ4’ flesh compared to ‘DH’. The upstream metabolites of naringenin such as calycosin, rotenone, and glycitein were significantly higher in ‘XJ4’ flesh, while there was no difference for these metabolites in peel analysis. The results of most of downstream differential metabolites were similar to what we found in peel comparison. Apigenin, 3′-*O*-methylluteolin, and genistein 7-*O*-glucoside were significantly increased in ‘XJ4’ flesh. The anthocyanins components cyanidin 3-*O*-glucoside, and cyanidin 3,5-*O*-diglucoside were significantly increased in ‘XJ4’ flesh compared to ‘DH’. Naringenin was detected significantly increased in ‘XJ4’ flesh compared to ‘DH’ but not in peel. As mentioned, quercetin and laricitrin that were found significantly higher in the peel of ‘XJ4’ compared to ‘DH’, but not in the flesh. In addition, syringetin was the only metabolite that had significantly higher level in ‘DH’ flesh. 

Here, it was obvious that significantly higher levels of metabolites in ‘XJ4’ flesh compared to ‘DH’ flesh were found in both early and late biosynthetic genes regulated domain. This was different from the comparison between ‘DH’ peel and ‘XJ4’ peel, which showed significantly higher levels of metabolites in ‘XJ4’ peel compared to ‘DH’ peel only existed in late biosynthetic genes-regulated domain. As a result, in ‘XJ4’ peel the decrease of upstream metabolites in the early biosynthetic genes regulated domain might cause the final anthocyanins content decrease. This might explain why the total anthocyanins content in ‘XJ4’ peel was decreased largely from young and developmental stages to mature stage, while the flesh anthocyanins were more stable through all three stages (Table 1).

In addition, it’s necessary to note that the huge accumulations of anthocyanins in peel and flesh such as pelargonidin 3-*O*-β-d-glucoside and cyanidin 3-*O*-malonylhexoside were detected in ‘XJ4’ compared to ‘DH’ (Table 3), but they were not annotated by KEGG into the corresponding pathway. The huge accumulations of cyanidin 3,5-*O*-diglucoside (cyanin), cyanidin 3-*O*-rutinoside (keracyanin), and cyanidin 3-*O*-glucoside were detected in ‘XJ4’ and at the same time annotated by KEGG into the anthocyanins biosynthesis pathway. What’s more, the accumulation of apigenin, naringenin and genistein 7-*O*-glucoside were detected in ‘XJ4’ and annotated by KEGG into the isoflavonoid biosynthesis pathway. Therefore, the accumulation of these six significantly differential metabolites in ‘XJ4’ resulted in the changes of anthocyanins biosynthesis pathway and isoflavonoid biosynthesis pathway. These significantly differential metabolites worked together and further regulated the peel and flesh of ‘XJ4’ to remain purple red, highest total phenols content, highest anthocyanins content, and higher free radicals scavenging ability. 

## 3. Conclusions

In this study we found generally the DPPH radical scavenging rate was positively related to anthocyanins content in four phenotypically different red-fleshed apple cultivars ‘XJ4’, ‘QN-5’, ‘DH’ and ‘HX1’. Among these four cultivars, ‘XJ4’ and ‘DH’ had the biggest difference in the aspects of fruit phenotype, anthocyanins content, and antioxidant activity (mainly DPPH) at mature stage. Therefore, in order to unveil the specific details and mechanism of anthocyanins accumulation in peel and flesh of these two cultivars, the differential flavonoids metabolites in peel and flesh of ‘XJ4’ and ‘DH’ were compared. We found pelargonidin 3-*O*-β-d-glucoside and cyanidin-3-*O*-malonylhexoside were the most abundant metabolites in XJ4PM and XJ4FM when compared to DHPM and DHFM. When ‘XJ4’ flesh compared to ‘DH’ flesh, the significantly increased metabolites were detected in both early biosynthetic genes regulated domain (such as calycosin, rotenone, glycitein and butin) and late biosynthetic genes regulated domain (such as naringin, apigenin and genistein 7-O-glucoside). However, in the peel comparison, the significantly higher levels of metabolites in ‘XJ4’ peel only existed in late biosynthetic genes regulated domain. The flesh anthocyanins were more stable through all three stages, which might because the significantly higher levels of metabolites in ‘XJ4’ flesh existed in both early and late biosynthetic genes regulated domain.

## 4. Materials and Methods

### 4.1. Plant Materials and Sampling

The four different phenotypic red-fleshed apple cultivars ‘XJ4’, ‘QN-5’, ‘DH’ and ‘HX1’ were grafted on *Malus Robusta* for 8 years at the experimental farm of Qingdao Agricultural University (Qingdao, China). Fruits were harvested on May 18 (30 days after anthesis, named as young fruit stage), July 12 (90 days after anthesis, named as developmental stage), and August 30 (140 days after anthesis, named as mature stage), respectively. The peel and flesh of fruits were sampled separately and freeze-dried in liquid nitrogen, lyophilized and then transferred into −80 °C freezer for further use.

### 4.2. Extraction and Determination of Total Anthocyanins Content

The total anthocyanins from red-fleshed apple were extracted with methanol-HCl (99:1, v/v) at the ratio of 1:10 (w/v, fruit tissues to extraction buffer) under dark for 15 h. The supernatant was subsequently filtered with a 0.45 um membrane and stored at −4 °C. Total anthocyanins content was determined by pH differential method [40]. The amount of 1 mL of the anthocyanins extract was added into 9 mL of sodium acetate buffer (0.4 mol/L, pH 4.5) and potassium chloride buffer (0.025 mol/L, pH 1.0), respectively. The absorbance of each mixture was detected at 510 nm and 700 nm using a spectrophotometer after incubation for 1 h at room temperature. Absorbance (A) of each sample was calculated by the equation:

A = (A510 − A700) _pH 1.0_ − (A510 − A700) _pH 4.5_, (1)

Total anthocyanins content (cyanidin 3-*O*-glucoside equivalents, mg·kg^−1^ FW) = A × MW × DF/ (ε × W). (2)

where MW (449.2) means the molecular weight of cyanidin 3-*O*-glucoside, DF means the dilution factor, ε (26,900) means the molar absorptivity of cyanidin 3-*O*-glucoside, and W means the fresh weight of each sample.

### 4.3. Determination of Total Phenols Content

The total phenol content was determined by Folin-Ciocalteu method, as modified by Cai [41]. The Folin-Ciocalteu’s phenol reagent was purchased from Beijing Solarbio Science and Technology Co., Ltd. (Beijing, China). Different concentrations of gallic acid solution was mixed with 10% sodium carbonate solution in a 5 mL volume system. The absorbance of mixture was detected by spectrophotometer at 765 nm after incubation at 50 °C for 1 h. Total phenol content was calculated by the equation:

Total phenols content (gallic acid equivalents, mg·kg^−1^ FW) = C × V × N/m. (3)

where C means the concentration calculated from standard curve, V means final volume of sample solution, N means dilution times, and m means the weight of sample.

### 4.4. Scavenging Capacity of DPPH Radical

The method of detecting scavenging capacity of DPPH radical was slightly modified from He [42]. The sample group consisted of 2 mL extract and 2 mL DPPH (0.2 mmol/L), DPPH was purchased from Shanghai Solarbio Science and Technology Co., Ltd. The blank group consisted of 2 mL extract and 2 mL ethanol. The group without sample consisted of 2 mL DPPH and 2 mL ethanol. After incubation at room temperature for 30 min in the dark, the absorbance of three groups was detected at 517 nm. The scavenging rate was calculated using the following equation:

Scavenging rate (%) = [1 − (As − Ab)/Aw] × 100%(4)

where As means the absorbance of sample group, Ab means the absorbance of blank group, and Aw means the absorbance of the group without sample.

### 4.5. Scavenging Capacity of ·OH

The assay of ·OH-scavenging was conducted following the method modified from Ma [43]. The amount of 1.5 mL phenanthrene solution (5 mmol/L) was added into 9 mL PBS buffer (0.01 M, pH 7.4). Totally 1 mL FeSO4 solution (7.5 mmol/L) was added into the mixture. Afterwards, 2.5 mL distilled water was injected into the injured group and uninjured group after adding 2.5 mL red-fleshed apple extract into the sample group. Ultimately, 1 mL 1% H_2_O_2_ solution was added into the injured group and sample group after 1 mL distilled water was put into uninjured group. The absorbance of three groups was detected at 536 nm after incubation at 37 °C for 1 h. The scavenging rate of ·OH was calculated with the equation:

Scavenging rate (%) = (As − Ai)/(Au − Ai) × 100%(5)

where As means the absorbance of sample group, Ai means the absorbance of injured group, and Au means the absorbance of uninjured group.

### 4.6. Scavenging Rate of O_2_·-

The modified method of pyrogallol autoxidation was used to detect scavenging rate of O_2_·- [44]. The amount of 4.5 mL Tris-HCl buffer (pH 8.0, 0.05 mol/L) was added into blank group and sample group, respectively. Totally 0.1 mL extract and 0.1 mL distilled water was added into blank group and sample group. Then, 0.4 mL pyrogallol (2.5 mmol/L) was introduced into two groups, respectively. After incubation at 25 °C for 5 min, two drops of HCl (8.0 mol/L) was used to end the reaction. The absorbance was detected at 325 nm. Scavenging rate of O_2_ was calculated by the equation:

Scavenging rate (%) = (Ab – As)/Ab × 100%(6)

where Ab means the absorbance of blank group and As means the absorbance of sample group.

### 4.7. Sample Preparation and Extraction for Metabolites Study

The freeze-dried peel and flesh of ‘XJ4’ and ‘DH’ at mature stage were crushed into powder using a mixer mill (MM400, Retsch, Shanghai, China) with a zirconia bead for 1.5 min at 30 Hz. A total of 100 mg powder of samples was weighted and extracted overnight at 4 °C with 1.0 mL 70% aqueous methanol. The extract was centrifuged at 10,000× *g* for 10 min and filtrated before LC-MS analysis. The samples were divided into two comparison groups, which were ‘DH’ Flesh Metabolites vs. ‘XJ4’ Flesh Metabolites (DHFM vs. XJ4FM), ‘DH’ Peel Metabolites vs. ‘XJ4’ Peel Metabolites (DHPM vs. XJ4PM). DHFM and DHPM were the control group in DHFM vs. XJ4FM, DHPM vs. XJ4PM respectively. The extract of peel and flesh from red-fleshed apple was absorbed (CNWBOND Carbon-GCB SPE cartridge, 250 mg, 3 mL; ANPEL, Shanghai, China) and filtered (SCAA-104, 0.22 μm pore size; ANPEL) before LC-MS analysis.

### 4.8. Metabolite Separation, Identification and Quantification

The sample analysis instruments system mainly consists of an Ultra Performance Liquid Chromatography system (UPLC, CBM30A Shimpack UFLC, Shimadzu, Kyoto, Japan; http://www.shimadzu.com.cn/) and a tandem mass spectrometry (MS/MS) instrument (6500 QTRAP, Applied Biosystems, AB Sciex, Waltham, MA, USA; http://www.appliedbiosystems.com.cn/). In total 2 µL of sample were injected into the HPLC system equipped with a C_18_ column (ACQUITY UPLC HSS T3, 1.8 µm, 2.1 mm × 100 mm, Waters, Milford, MA, USA). Ultra-pure water with 0.04% acetic acid was used as mobile phase A and acetonitrile with 0.04% acetic acid was used as mobile phase B. The gradient program was 100:0 v/v at 0 min, 5:95 v/v at 11.0 min, 5:95 v/v at 12.0 min, 95:5 v/v at 12.1 min, 95:5 v/v at 15.0 min. The flow rate was 0.40 mL/min and the column temperature was 40 °C. The effluent was connected to electrospray ionization (ESI)-triple quadrupole-linear ion trap-MS/MS system (6500 Q TRAP). The ion source was turbo spray and source temperature was maintained at 500 °C. The ion spray voltage (IS) was 5500 V and the collision gas (CAD) was high. In addition, the ion source gas I (GSI), gas II (GSII), and curtain gas (CUR) were set at 55.0, 60.0, and 25.0 psi, respectively. Triple quadrupole (QQQ) scans were acquired as MRM experiments with collision gas (nitrogen) set at 5 psi. The high performance liquid chromatography (HPLC) effluent was connected to the Applied Biosystems 6500 Q TRAP electrospray ionization (ESI)-triple quadrupole-linear ion trap-MS/MS system. A specific set of multiple-reaction monitoring (MRM) transitions was monitored for each period. Metabolite identification was based on MWDB (metware database), MassBank (http://www.massbank.jp/), KNAPSAcK (http://kanaya.naist.jp/KNApSAcK/), HMDB (http://www.hmdb.ca/), METLIN (http://metlin.scripps.edu/index.php), MoTo DB (http://www.ab.wur.nl/moto/). The thresholds of variable importance in projection (VIP) ≥1 and fold change ≥2 or ≤0.5 were set to filter metabolites with significant differences in content.

### 4.9. Statistical Analysis

All the experiments were conducted with three replicates. The data was presented as means ± SE. Significant differences among groups were tested using one-way ANOVA test. Photoshop CS6 (Adobe, San Jose, CA, USA) analysis software and Origin 9.0 software (Northampton, MA, USA) were used to test multiple comparisons and plot charts.

## Figures and Tables

**Table 1:** Total anthocyanins and total phenols content in peel and flesh of four phenotypically different red-fleshed apples at young, developmental and mature stages.

Variety of Red-Fleshed Apple	TAC		TPC	
	Peel	Flesh	Peel	Flesh
**Young stage**				
XJ4	1702.6 ± 75.4a	516.3 ± 35.6a	8114.4 ± 152.4a	3724.4 ± 359.9b
QN-5	461.3 ± 20.6b	355.1 ± 118.4b	3233.6 ± 675.8cd	2522.6 ± 935.3cd
DH	170.6 ± 47.5d	99.9 ± 46.1c	5277.3 ± 1229.1b	3269.2 ± 288.9bc
HX1	109.1 ± 25.9de	66.5 ± 1.9cd	9082.3 ± 717.1a	5346.1 ± 450.8a
**Developmental stage**				
XJ4	1506 ± 101.4a	631 ± 38.7a	5460.3 ± 532.3b	2397.1 ± 699.5cd
QN-5	253.1 ± 46.9c	361 ± 45.5b	2724.6 ± 247.7cde	1198.8 ± 181.2e
DH	35 ± 15f	54.2 ± 34.5cd	3171.2 ± 528.1cd	1127.1 ± 163.1e
HX1	111 ± 71.1de	71.5 ± 11.7c	2636.5 ± 154.5cde	1974.9 ± 373.7d
**Mature stage**				
XJ4	441 ± 76.3b	401 ± 13.5b	3333.9 ± 432.2c	2283.6 ± 520.2cd
QN-5	93.4 ± 3.3de	76.6 ± 16.1c	1239.6 ± 481.4f	327.6 ± 54.4f
DH	12.1 ± 3.4f	24.3 ± 6.7d	2069.3 ± 276de	1010.2 ± 280.6e
HX1	80.1 ± 13.4e	57.3 ± 1.1cd	2691.9 ± 239.7cde	1598.8 ± 287.3de

TAC means Total Anthocyanins Content (cyanidin 3-*O*-glucoside equivalents, mg·kg^−1^); TPC means Total Phenols Content (gallic acid equivalents, mg·kg^−1^). Within samples, the significant difference (*p* < 0.05) is represented by different letters (a, b, c, d, e and f).

**Table 2:** In vitro antioxidant activity of anthocyanins extract from peel and flesh of four phenotypically different red-fleshed apples at young, developmental and mature stages.

Variety of Red-Fleshed Apple	Scavenging Rate of DPPH/%		Scavenging Rate of OH/%		Scavenging Rate of O_2_*·*-/%		Dilution Ratio	
	Peel	Flesh	Peel	Flesh	Peel	Flesh	Peel	Flesh
**Young stage**								
XJ4	57.7 ± 2.8de	80.9 ± 7.8ab	14.3 ± 1.5e	31.4 ± 8.0c	22.1 ± 1.0d	12 ± 4.6f	34.0	10.3
QN-5	88.1 ± 0.8a	85.7 ± 1.0ab	46.1 ± 2.9c	49.5 ± 8.1b	11.1 ± 1.3e	18.4 ± 2.9ef	9.2	7.1
DH	84.7 ± 6.1ab	81.2 ± 3.8ab	49.2 ± 5.6bc	38.5 ± 1.7c	27.2 ± 0.7cd	27.6 ± 0.7cde	3.4	2.0
HX1	70.9 ± 1.7bc	81.4 ± 3.6ab	81.5 ± 7.3a	51.6 ± 11.2b	21.7 ± 0.8d	15.3 ± 1.6ef	2.1	1.3
**Developmental stage**								
XJ4	90.7 ± 9.0a	84.5 ± 1.6ab	33.7 ± 3.1d	37.3 ± 3.5c	47.7 ± 12.6ab	31.7 ± 2.4abc	30.1	12.6
QN-5	94.7 ± 0.2a	94.6 ± 0.2ab	43 ± 4.0cd	54 ± 3.9b	52.7 ± 4.8a	25.9 ± 10.1cde	5.0	7.2
DH	48.5 ± 10.2e	80 ± 3.2b	40.1 ± 6.0cd	13.8 ± 3.1e	29.4 ± 7.4cd	21.1 ± 4.1def	−1.4	1.0
HX1	65.8 ± 1.3cd	83.5 ± 0.7ab	41.8 ± 3.6cd	34.2 ± 6.1c	31.1 ± 3.2bcd	17.1 ± 3.1ef	2.2	1.4
**Mature stage**								
XJ4	90.6 ± 2.1a	97.1 ± 1.2a	59.4 ± 8.6b	75.1 ± 0.71a	39.7 ± 2.7abc	39.9 ± 2.5ab	8.8	8.0
QN-5	93.3 ± 2.5a	94.7 ± 0.4ab	2.4 ± 1.0g	1.5 ± 0.4g	38 ± 4.8abc	43.3 ± 4.8a	1.8	1.5
DH	21.4 ± 4.5f	46.5 ± 3.0c	48.9 ± 8.4bc	20.1 ± 5.7d	39 ± 3.2abc	47.1 ± 4.7a	−4.6	−2
HX1	82.7 ± 1.6ab	88.3 ± 0.9ab	15.2 ± 3.0e	4.3 ± 0.1f	32.2 ± 2.5bcd	36.8 ± 3.1abc	1.6	1.1
VC	3.6 ± 1.6g	3.6 ± 1.6d	9.1 ± 0.8f	9.1 ± 0.8e	19.1 ± 9.0d	19.1 ± 9.0ef		

The significant difference (*p* < 0.05) is represented by different letters (a, b, c, d, e and f).

**Table 3:** The extremely significantly different metabolites in DHPM vs. XJ4PM and DHFM vs. XJ4FM.

Combination Name	Metabolite Name	Content		Fold Change (XJ4PM/DHPM; XJ4FM/DHFM)	VIP	Grouping of Specific Metabolites
		DH	XJ4			
**ANTHOCYANINS**						
DHPM vs. XJ4PM	Pelargonidin 3-*O*-β-d-glucoside (callistephin chloride)	9.00E + 00	3.14E + 07	3.49E + 06	2.36	XJ4PM
	Cyanidin 3-*O*-malonylhexoside	9.00E + 00	1.32E + 06	1.47E + 05	2.1	XJ4PM
	Malvidin 3-acetyl-5-diglucoside	9.00E + 00	2.56E + 05	2.84E + 04	1.93	XJ4PM
	Cyanidin 3-*O*-rutinoside (Keracyanin)	9.00E + 00	2.40E + 05	2.67E + 04	1.94	XJ4PM
	Pelargonidin 3-*O*-malonylhexoside	9.00E + 00	1.63E + 04	1.82E + 03	1.65	XJ4PM
DHFM vs. XJ4FM	Pelargonidin 3-*O*-β-d-glucoside	9.00E + 00	6.78E + 07	7.54E + 06	2.16	XJ4FM
	Cyanidin 3-*O*-malonylhexoside	9.00E + 00	1.56E + 06	1.74E + 05	1.89	XJ4FM
	Malvidin 3-acetyl-5-diglucoside	9.00E + 00	1.06E + 06	1.17E + 05	1.86	XJ4FM
	Peonidin *O*-hexoside	9.00E + 00	7.50E + 05	8.33E + 04	1.83	XJ4FM
	Peonidin 3-*O*-glucoside chloride	9.00E + 00	7.12E + 05	7.91E + 04	1.83	XJ4FM
	Pelargonidin 3-*O*-malonylhexoside	9.00E + 00	2.49E + 04	2.76E + 03	1.53	XJ4FM
	Rosinidin *O*-hexoside	7.00E + 04	9.00E + 00	1.29E-04	1.62	DHFM
**FLAVONES**						
DHPM vs. XJ4PM	Selgin *O*-hexosyl-*O*-hexoside	9.00E + 00	3.09E + 06	3.43E + 05	2.17	XJ4PM
	Tricin *O*-saccharic acid	9.00E + 00	3.02E + 06	3.36E + 05	2.16	XJ4PM
	6-C-hexosyl-hesperetin *O*-hexoside	9.00E + 00	2.41E + 06	2.68E + 05	2.14	XJ4PM
	Luteolin 3’,7-di-*O*-glucoside	9.00E + 00	1.26E + 06	1.39E + 05	2.09	XJ4PM
	*O*-methylChrysoeriol 5-*O*-hexoside	9.00E + 00	1.09E + 06	1.22E + 05	1.38	XJ4PM
	*O*-methylChrysoeriol 7-*O*-hexoside	9.00E + 00	1.09E + 06	1.21E + 05	1.37	XJ4PM
	C-hexosyl-apigenin *O*-hexosyl-*O*-hexoside	9.00E + 00	8.63E + 05	9.59E + 04	2.05	XJ4PM
	Eriodictiol 6-C-hexoside 8-C-hexoside-*O*-hexoside	9.00E + 00	5.88E + 05	6.54E + 04	2.02	XJ4PM
	Hesperetin C-hexosyl-*O*-hexosyl-*O*-hexoside	9.00E + 00	5.85E + 05	6.50E + 04	2.02	XJ4PM
	C-hexosyl-isorhamnetin *O*-hexoside	9.00E + 00	1.67E + 05	1.86E + 04	1.9	XJ4PM
	Tricin 4′-*O*-(syringyl alcohol) ether 5-*O*-hexoside	9.00E + 00	1.55E + 05	1.73E + 04	1.89	XJ4PM
	Morin	9.00E + 00	7.46E + 05	8.29E + 04	2.04	XJ4PM
	Persicoside	9.00E + 00	5.93E + 05	6.59E + 04	2.02	XJ4PM
	Chrysoeriol 8-C-hexoside	9.00E + 00	4.92E + 04	5.47E + 03	1.25	XJ4PM
	Apigenin	9.00E + 00	4.38E + 04	4.87E + 03	1.22	XJ4PM
	Acacetin	9.00E + 00	3.31E + 04	3.68E + 03	1.66	XJ4PM
	Tricin 5-*O*-rutinoside	9.00E + 00	1.14E + 04	1.26E + 03	1.15	XJ4PM
	Baicalein-7-*O*-glucuronide (Baicalin)	5.51E + 04	9.00E + 00	1.63E-04	1.79	DHPM
	Diosmin	2.21E + 06	9.00E + 00	4.08E-06	2.11	DHPM
	Chrysoeriol 7-*O*-rutinoside	9.30E + 05	9.00E + 00	9.67E-06	2.03	DHPM
DHFM vs. XJ4FM	6-C-hexosyl-hesperetin *O*-hexoside	9.00E + 00	7.26E + 06	8.06E + 05	2	XJ4FM
	Luteolin 3’,7-di-*O*-glucoside	9.00E + 00	3.64E + 06	4.04E + 05	1.95	XJ4FM
	Selgin *O*-hexosyl-*O*-hexoside	9.00E + 00	1.98E + 06	2.20E + 05	1.91	XJ4FM
	8-C-hexosyl-hesperetin *O*-hexoside	9.00E + 00	6.70E + 05	7.45E + 04	1.82	XJ4FM
	Eriodictiol 6-C-hexoside 8-C-hexoside-*O*-hexoside	9.00E + 00	6.06E + 05	6.74E + 04	1.81	XJ4FM
	Hesperetin C-hexosyl-*O*-hexosyl-*O*-hexoside	9.00E + 00	4.05E + 05	4.50E + 04	1.78	XJ4FM
	Limocitrin *O*-hexoside	9.00E + 00	3.43E + 05	3.81E + 04	1.77	XJ4FM
	Chrysoeriol 8-C-hexoside	9.00E + 00	2.49E + 05	2.76E + 04	1.74	XJ4FM
	Chrysoeriol 6-C-hexoside 8-C-hexoside-*O*-hexoside	9.00E + 00	2.28E + 05	2.53E + 04	1.73	XJ4FM
	Butin	9.00E + 00	1.82E + 05	2.02E + 04	1.71	XJ4FM
	Eriocitrin	9.00E + 00	7.77E + 04	8.63E + 03	1.64	XJ4FM
	Tricin *O*-saccharic acid	9.00E + 00	5.43E + 04	6.03E + 03	1.6	XJ4FM
	*O*-methylChrysoeriol 7-*O*-hexoside	9.00E + 00	4.73E + 04	5.26E + 03	1.59	XJ4FM
	Tricin 5-*O*-rutinoside	9.00E + 00	4.45E + 04	4.94E + 03	1.59	XJ4FM
	*O*-methylChrysoeriol 5-*O*-hexoside	9.00E + 00	3.99E + 04	4.43E + 03	1.58	XJ4FM
	Apigenin	9.00E + 00	1.39E + 04	1.55E + 03	1.03	XJ4FM
	Chrysoeriol	9.00E + 00	7.42E + 03	8.25E + 02	1.41	XJ4FM
	Chrysoeriol 7-*O*-rutinoside	1.68E + 04	9.00E + 00	5.37E-04	1.04	DHFM
	C-hexosyl-luteolin *O*-sinapic acid	2.41E + 04	9.00E + 00	3.74E-04	1.52	DHFM
	Luteolin C-hexoside	2.42E + 04	9.00E + 00	3.72E-04	1.53	DHFM
	6-C-hexosyl luteolin *O*-pentoside	3.15E + 04	9.00E + 00	2.86E-04	1.09	DHFM
	Diosmin	3.20E + 04	9.00E + 00	2.81E-04	1.08	DHFM
	Luteolin *O*-sinapoylhexoside	4.34E + 04	9.00E + 00	2.07E-04	1.58	DHFM
	3-*O*-Acetylpinobanksin	4.79E + 04	9.00E + 00	1.88E-04	1.53	DHFM
**FLAVONOLS**						
DHPM vs. XJ4PM	Quercetin	9.00E + 00	5.59E + 05	6.21E + 04	2.02	XJ4PM
	Laricitrin	9.00E + 00	6.04E + 04	6.71E + 03	1.8	XJ4PM
	Isorhamnetin	9.00E + 00	2.26E + 04	2.51E + 03	1.7	XJ4PM
	Rhamnetin (7-*O*-methxyl quercetin)	9.00E + 00	3.26E + 03	3.62E + 02	1.47	XJ4PM
	Kaempferol 3-*O*-rutinoside (Nicotiflorin)	1.16E + 06	9.00E + 00	7.77E-06	2.08	DHPM
DHFM vs. XJ4FM	Syringetin 3-*O*-hexoside	9.00E + 00	4.68E + 05	5.20E + 04	1.79	XJ4FM
	Rhamnetin	9.00E + 00	5.00E + 03	5.56E + 02	1.36	XJ4FM
	Isorhamnetin 3-*O*-neohesperidoside	1.08E + 04	9.00E + 00	8.33E-04	1.01	DHFM
	Kaempferol 3-*O*-robinobioside (Biorobin)	3.84E + 04	9.00E + 00	2.34E-04	1.1	DHFM
	Syringetin	6.33E + 04	9.00E + 00	1.42E-04	1.6	DHFM
**FLAVANONES**						
DHPM vs. XJ4PM	Hesperetin *O*-malonylhexoside	9.00E + 00	1.22E + 05	1.35E + 04	1.87	XJ4PM
DHFM vs. XJ4FM	Naringenin	9.00E + 00	1.92E + 05	2.13E + 04	1.72	XJ4FM
	Hesperetin *O*-malonylhexoside	9.00E + 00	1.55E + 05	1.72E + 04	1.7	XJ4FM
	Naringenin 7-*O*-neohesperidoside (Naringin)	9.00E + 00	9.60E + 04	1.07E + 04	1.66	XJ4FM
	Hesperetin *O*-Glucuronic acid	9.00E + 00	4.25E + 04	4.72E + 03	1.58	XJ4FM
**ISOFLAVONES**						
DHPM vs. XJ4PM	Genistein 7-*O*-Glucoside (Genistin)	9.00E + 00	1.87E + 05	2.08E + 04	1.92	XJ4PM
DHFM vs. XJ4FM	Rotenone	9.00E + 00	4.27E + 04	4.74E + 03	1.58	XJ4FM
	Genistein 7-*O*-Glucoside	9.00E + 00	2.79E + 04	3.10E + 03	1.08	XJ4FM
	Glycitein	9.00E + 00	9.10E + 03	1.01E + 03	1.43	XJ4FM
	Calycosin	9.00E + 00	6.75E + 03	7.50E + 02	1.4	XJ4FM
**POLYPHENOLS**						
DHFM vs. XJ4FM	Gallocatechin-gallocatechin	9.00E + 00	2.90E + 07	3.23E + 06	2.11	XJ4FM

## Supplementary Materials

Figure S1 Principle component analysis (PCA) score plot, orthogonal partial least squares-discriminant analysis (OPLS-DA) score plot and permutation test in four comparison groups. (A,B) PCA in DHPM vs. XJ4PM and DHFM vs. XJ4FM. (C,D) OPLS-DA in DHPM vs. XJ4PM and DHFM vs. XJ4FM. (E,F) Permutation test in DHPM vs. XJ4PM and DHFM vs. XJ4FM. Table S1 The differential metabolites in both DHPM vs. XJ4PM and DHFM vs. XJ4FM.

## Abbreviations

2HID	2-Hydroxyisoflavanone dehydratase
2HIFD	2-Hydroxyisoflavanone dehydratase
6’DCS	6′-Deoxychalcone synthase
ANR	Anthocyanidin reductase
ANS	Anthocyanidin synthase
CHI	Chalcone isomerase
CHS	Chalcone synthase
DFR	Dihydroflavonol 4-reductase
DH	Daihong
DHFM	Flesh of ‘DH’ at mature stage
DHPM	Peel of ‘DH’ at mature stage
ESI	Electrospray ionization
F3GRT	Flavonol-3-*O*-glucoside L-rhamnosyltransferase
F3GT	Flavonol 3-*O*-glucosyltransferase
F3H	Flavanone 3-hydroxylase
F3’5’H	Flavanoid 3’,5′-hydroxylase
F3’5’MT	Flavonoid 3’,5′-methyltransferase
F3’H	Flavoniod 3′-hydroxylose
F3’H	Flavonoid 3′-hydroxylase
F3’M	Flavonoid 3′-monooxygenase
FLS	Flavonol synthase
FSI	Flavone synthase I
HPLC	High performance liquid chromatography
HX1	Hongxun No.1
IF3’H	Isoflavone 3′-hydroxylase
IFS	Isoflavonoid synthase
K4MT	Kaempferol 4-*O*-methyltransferase
L3’M	Luteolin 3′-*O*-methyltransferase
LAR	Leucoanthocyanin reductase
LCR	Leucocyanidin reductase
MRM	Multiple-reaction monitoring
N7GT	Naringenin 7-*O*-glucosyltransferase
QN-5	Qingnong No.5
SAM	2,7,4′-trihydroxyisoflavanone 4′-*O*-methyltransferase
UAGT	UDP-glucose-anthocyanin 5-*O*-glucosyltransferase
UCRGT	UDP-glucose-cyanidin-d-rhamnosyl-glucosyltransferase
UFGT	UDP glucose-flavonoid 3-*O*-glcosyl-transferase
URT	UDP-rhamnosetransferase
VIP	Variable importance in projection
