# Supplementary material for: Analysis of Antioxidant Activity and Flavonoids Metabolites in Peel and Flesh of Red-Fleshed Apple Varieties
Source: Molecules. 2020 Apr 23;25(8):1968. doi: 10.3390/molecules25081968 (PMC7221745; doi:10.3390/molecules25081968)
Supplement: Supplementary file 1 [file molecules-25-01968-s001.pdf]

molecules

MDPI

Supplementary

Analysis of Antioxidant Activity and Flavonoids Metabolites in Peel and Flesh of Red-Fleshed Apple Varieties

Xiang Zhang [1,2,†], Jihua Xu [1,3,†], Zhaobo Xu [4], Xiaohong Sun [1,3], Jun Zhu [1,2] and Yugang Zhang [1,2,*]

[1] Qingdao Key Laboratory of Genetic Development and Breeding in Horticultural Plants, Qingdao Agricultural University, Qingdao 266109, China; 18306391375@163.com (X.Z.); xujihua@qau.edu.cn (J.X.); mingsun9887@163.com (X.S.); junzhu@qau.edu.cn (J.Z.)

[2] College of Horticulture, Qingdao Agricultural University, Qingdao 266109, China

[3] College of Life Sciences, Qingdao Agricultural University, Qingdao 266109, China

[4] Qingdao Agriculture Technology Extension Center, Qingdao 266071, China; 18661683886@163.com (Z.X.)

* Correspondence: ygzhang@qau.edu.cn; Tel.: + 86-0532-589-57740

† These authors contributed equally to this work.

Received: 17 March 2020; Accepted: 21 April 2020; Published:

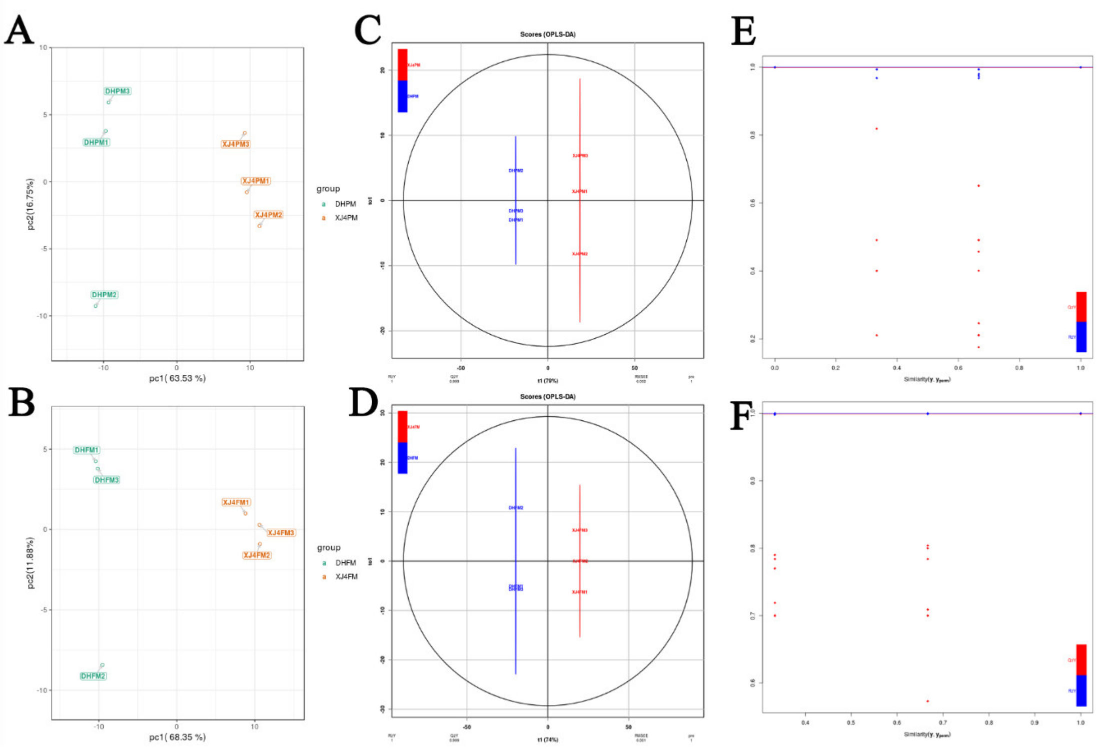

Figure S1. Principle component analysis (PCA) score plot, orthogonal partial least squares-discriminant analysis (OPLS-DA) score plot and permutation test in four comparison groups. (**A**,**B**) PCA in DHPM vs. XJ4PM and DHFM vs. XJ4FM. (**C**,**D**) OPLS-DA in DHPM vs. XJ4PM and DHFM vs. XJ4FM. (**E**,**F**) Permutation test in DHPM vs. XJ4PM and DHFM vs. XJ4FM.

Table S1. The differential metabolites in both DHPM vs. XJ4PM and DHFM vs. XJ4FM.

Combination name	Metabolite name	Content		Fold change (XJ4PM/DHPM; XJ4FM/DHFM)	VIP	Grouping of specific metabolites
		DH	XJ4			
**ANTHOCYANIN**						
DHPM vs. XJ4PM	Pelargonidin 3-*O*-beta-D-glucoside（Callistephin chloride)	9.00E + 00	3.14E + 07	3.49E + 06	2.36	XJ4PM
	Cyanidin 3-*O*-malonylhexoside	9.00E + 00	1.32E + 06	1.47E + 05	2.1	XJ4PM
	Malvidin 3-acetyl-5-diglucoside	9.00E + 00	2.56E + 05	2.84E + 04	1.93	XJ4PM
	Cyanidin 3-*O*-rutinoside (Keracyanin)	9.00E + 00	2.40E + 05	2.67E + 04	1.94	XJ4PM
	Pelargonidin 3-*O*-malonylhexoside	9.00E + 00	1.63E + 04	1.82E + 03	1.65	XJ4PM
	Cyanidin *O*-syringic acid	2.69E + 05	1.20E + 07	4.47E + 01	1.19	XJ4PM ＆DHPM
	Peonidin *O*-hexoside	4.15E + 05	1.66E + 07	4.00E + 01	1.17	XJ4PM ＆DHPM
	Peonidin 3-*O*-glucoside chloride	4.46E + 05	1.73E + 07	3.87E + 01	1.17	XJ4PM ＆DHPM
	Cyanidin 3-*O*-galactoside	2.77E + 05	8.51E + 06	3.07E + 01	1.13	XJ4PM ＆DHPM
	Cyanidin 3-*O*-glucoside (Kuromanin)	2.34E + 06	7.13E + 07	3.05E + 01	1.13	XJ4PM ＆DHPM
	Cyanidin 3,5-*O*-diglucoside (Cyanin)	1.15E + 06	3.19E + 07	2.78E + 01	1.11	XJ4PM ＆DHPM
DHFM vs. XJ4FM	Pelargonidin 3-*O*-beta-D-glucoside	9.00E + 00	6.78E + 07	7.54E + 06	2.16	XJ4FM
	Cyanidin 3-*O*-malonylhexoside	9.00E + 00	1.56E + 06	1.74E + 05	1.89	XJ4FM
	Malvidin 3-acetyl-5-diglucoside	9.00E + 00	1.06E + 06	1.17E + 05	1.86	XJ4FM
	Peonidin *O*-hexoside	9.00E + 00	7.50E + 05	8.33E + 04	1.83	XJ4FM
	Peonidin 3-*O*-glucoside chloride	9.00E + 00	7.12E + 05	7.91E + 04	1.83	XJ4FM
	Pelargonidin 3-*O*-malonylhexoside	9.00E + 00	2.49E + 04	2.76E + 03	1.53	XJ4FM
	Cyanidin 3,5-*O*-diglucoside	9.27E + 04	4.35E + 07	4.69E + 02	1.35	XJ4FM ＆DHFM
	Cyanidin *O*-syringic acid	6.76E + 04	1.38E + 07	2.04E + 02	1.25	XJ4FM ＆DHFM
	Cyanidin 3-*O*-glucoside	7.30E + 05	7.71E + 07	1.06E + 02	1.18	XJ4FM ＆DHFM
	Cyanidin 3-*O*-galactoside	9.42E + 04	9.76E + 06	1.04E + 02	1.17	XJ4FM ＆DHFM
	Peonidin 3-sophoroside-5-glucoside.	1.82E + 03	7.96E + 03	4.37E + 00	1.00	XJ4FM ＆DHFM
	Rosinidin *O*-hexoside	7.00E + 04	9.00E + 00	1.29E-04	1.62	DHFM
**FLAVONE**						
DHPM vs. XJ4PM	Selgin *O*-hexosyl-*O*-hexoside	9.00E + 00	3.09E + 06	3.43E + 05	2.17	XJ4PM
	Tricin *O*-saccharic acid	9.00E + 00	3.02E + 06	3.36E + 05	2.16	XJ4PM
	6-C-hexosyl-hesperetin *O*-hexoside	9.00E + 00	2.41E + 06	2.68E + 05	2.14	XJ4PM
	Luteolin 3′,7-di-*O*-glucoside	9.00E + 00	1.26E + 06	1.39E + 05	2.09	XJ4PM
	O-methylChrysoeriol 5-*O*-hexoside	9.00E + 00	1.09E + 06	1.22E + 05	1.38	XJ4PM

	O-methylChrysoeriol 7-*O*-hexoside	9.00E + 00 1.09E + 06	1.21E + 05	1.37	XJ4PM
	C-hexosyl-apigenin *O*-hexosyl-*O*-hexoside	9.00E + 00 8.63E + 05	9.59E + 04	2.05	XJ4PM
	Morin	9.00E + 00 7.46E + 05	8.29E + 04	2.04	XJ4PM
	Persicoside	9.00E + 00 5.93E + 05	6.59E + 04	2.02	XJ4PM
	Eriodictiol 6-C-hexoside 8-C-hexoside-*O*-hexoside	9.00E + 00 5.88E + 05	6.54E + 04	2.02	XJ4PM
	Hesperetin C-hexosyl-*O*-hexosyl-*O*-hexoside	9.00E + 00 5.85E + 05	6.50E + 04	2.02	XJ4PM
	C-hexosyl-isorhamnetin *O*-hexoside	9.00E + 00 1.67E + 05	1.86E + 04	1.90	XJ4PM
	Tricin 4′-*O*-(syringyl alcohol) ether 5-*O*-hexoside	9.00E + 00 1.55E + 05	1.73E + 04	1.89	XJ4PM
	Chrysoeriol 8-C-hexoside	9.00E + 00 4.92E + 04	5.47E + 03	1.25	XJ4PM
	Apigenin	9.00E + 00 4.38E + 04	4.87E + 03	1.22	XJ4PM
	Acacetin	9.00E + 00 3.31E + 04	3.68E + 03	1.66	XJ4PM
	Tricin 5-*O*-rutinoside	9.00E + 00 1.14E + 04	1.26E + 03	1.15	XJ4PM
	Selgin *O*-malonylhexoside	3.92E + 05 3.01E + 07	7.68E + 01	1.30	XJ4PM & DHPM
	Liquiritin	8.43E + 04 2.43E + 06	2.89E + 01	1.00	XJ4FM & DHFM
	Apigenin 5-*O*-glucoside	5.96E + 05 1.43E + 07	2.40E + 01	1.08	XJ4PM & DHPM
	Astilbin	3.85E + 04 7.91E + 05	2.06E + 01	1.05	XJ4PM & DHPM
	Eriodictiol C-hexosyl-*O*-hexoside	1.25E + 05 2.30E + 06	1.84E + 01	1.60	XJ4PM & DHPM
	Chrysoeriol	2.37E + 03 3.61E + 04	1.52E + 01	1.34	XJ4PM & DHPM
	Chrysoeriol 6-C-hexoside 8-C-hexoside-*O*-hexoside	5.59E + 05 4.47E + 04	8.00E-02	1.45	XJ4PM & DHPM
	Luteolin 8-C-hexosyl-*O*-hexoside	1.30E + 06 5.20E + 04	4.01E-02	1.01	XJ4PM & DHPM
	Baicalein-7-*O*-glucuronide (Baicalin)	5.51E + 04 9.00E + 00	1.63E-04	1.79	DHPM
	Chrysoeriol 7-*O*-rutinoside	9.30E + 05 9.00E + 00	9.67E-06	2.03	DHPM
	Diosmin	2.21E + 06 9.00E + 00	4.08E-06	2.11	DHPM
DHFM vs. XJ4FM	6-C-hexosyl-hesperetin *O*-hexoside	9.00E + 00 7.26E + 06	8.06E + 05	2.00	XJ4FM
	Luteolin 3′,7-di-*O*-glucoside	9.00E + 00 3.64E + 06	4.04E + 05	1.95	XJ4FM
	Selgin *O*-hexosyl-*O*-hexoside	9.00E + 00 1.98E + 06	2.20E + 05	1.91	XJ4FM
	8-C-hexosyl-hesperetin *O*-hexoside	9.00E + 00 6.70E + 05	7.45E + 04	1.82	XJ4FM
	Eriodictiol 6-C-hexoside 8-C-hexoside-*O*-hexoside	9.00E + 00 6.06E + 05	6.74E + 04	1.81	XJ4FM
	Hesperetin C-hexosyl-*O*-hexosyl-*O*-hexoside	9.00E + 00 4.05E + 05	4.50E + 04	1.78	XJ4FM
	Limocitrin *O*-hexoside	9.00E + 00 3.43E + 05	3.81E + 04	1.77	XJ4FM
	Chrysoeriol 8-C-hexoside	9.00E + 00 2.49E + 05	2.76E + 04	1.74	XJ4FM
	Chrysoeriol 6-C-hexoside 8-C-hexoside-*O*-hexoside	9.00E + 00 2.28E + 05	2.53E + 04	1.73	XJ4FM
	Butin	9.00E + 00 1.82E + 05	2.02E + 04	1.71	XJ4FM
	Eriocitrin	9.00E + 00 7.77E + 04	8.63E + 03	1.64	XJ4FM

	Tricin *O*-saccharic acid	9.00E + 00 5.43E + 04	6.03E + 03	1.60	XJ4FM
	O-methylChrysoeriol 7-*O*-hexoside	9.00E + 00 4.73E + 04	5.26E + 03	1.59	XJ4FM
	Tricin 5-*O*-rutinoside	9.00E + 00 4.45E + 04	4.94E + 03	1.59	XJ4FM
	O-methylChrysoeriol 5-*O*-hexoside	9.00E + 00 3.99E + 04	4.43E + 03	1.58	XJ4FM
	Apigenin	9.00E + 00 1.39E + 04	1.55E + 03	1.03	XJ4FM
	Chrysoeriol	9.00E + 00 7.42E + 03	8.25E + 02	1.41	XJ4FM
	Selgin 5-*O*-hexoside	5.45E + 04 2.81E + 06	5.15E + 01	1.20	XJ4FM & DHFM
	Apigenin *O*-hexosyl-*O*-rutinoside	5.41E + 03 1.27E + 05	2.36E + 01	1.30	XJ4FM & DHFM
	Naringenin C-hexoside	1.98E + 04 2.21E + 05	1.11E + 01	1.28	XJ4FM & DHFM
	Narcissoside	6.73E + 04 2.85E + 03	4.23E-02	1.23	XJ4FM & DHFM
	Chrysoeriol 7-*O*-rutinoside	1.68E + 04 9.00E + 00	5.37E-04	1.04	DHFM
	C-hexosyl-luteolin *O*-sinapic acid	2.41E + 04 9.00E + 00	3.74E-04	1.52	DHFM
	Luteolin C-hexoside	2.42E + 04 9.00E + 00	3.72E-04	1.53	DHFM
	6-C-hexosyl luteolin *O*-pentoside	3.15E + 04 9.00E + 00	2.86E-04	1.09	DHFM
	Diosmin	3.20E + 04 9.00E + 00	2.81E-04	1.08	DHFM
	Luteolin *O*-sinapoylhexoside	4.34E + 04 9.00E + 00	2.07E-04	1.58	DHFM
	3-*O*-Acetylpinobanksin	4.79E + 04 9.00E + 00	1.88E-04	1.53	DHFM
**FLAVONOL**					
DHPM vs. XJ4PM	Quercetin	9.00E + 00 5.59E + 05	6.21E + 04	2.02	XJ4PM
	Laricitrin	9.00E + 00 6.04E + 04	6.71E + 03	1.80	XJ4PM
	Isorhamnetin	9.00E + 00 2.26E + 04	2.51E + 03	1.70	XJ4PM
	Rhamnetin (7-*O*-methxyl quercetin)	9.00E + 00 3.26E + 03	3.62E + 02	1.47	XJ4PM
	Isorhamnetin *O*-acetyl-hexoside	7.88E + 04 6.68E + 06	8.47E + 01	1.33	XJ4PM & DHPM
	Quercetin-3,4′-*O*-di-beta-glucopyranoside	2.39E + 04 3.38E + 05	1.42E + 01	1.46	XJ4PM & DHPM
	Isorhamnetin 3-*O*-neohesperidoside	2.21E + 05 1.02E + 04	4.63E-02	1.03	XJ4PM & DHPM
	Quercetin 3-*O*-rutinoside (Rutin)	5.91E + 07 1.13E + 06	1.92E-02	1.13	XJ4PM & DHPM
	Quercetin 7-*O*-rutinoside	3.48E + 07 5.03E + 05	1.45E-02	1.17	XJ4PM & DHPM
	Kaempferol 3-*O*-robinobioside (Biorobin)	1.13E + 06 1.54E + 04	1.37E-02	1.66	XJ4PM & DHPM
	Kaempferol 3-*O*-rutinoside (Nicotiflorin)	1.16E + 06 9.00E + 00	7.77E-06	2.08	DHPM
DHFM vs. XJ4FM	Syringetin 3-*O*-hexoside	9.00E + 00 4.68E + 05	5.20E + 04	1.79	XJ4FM
	Rhamnetin	9.00E + 00 5.00E + 03	5.56E + 02	1.36	XJ4FM
	Myricetin 3-*O*-rhamnoside (Myricitrin)	1.93E + 05 1.21E + 07	6.28E + 01	1.11	XJ4FM & DHFM
	methylQuercetin *O*-hexoside	3.64E + 04 1.89E + 06	5.20E + 01	1.20	XJ4FM & DHFM
	Myricetin 3-*O*-galactoside	4.83E + 04 1.55E + 06	3.21E + 01	1.02	XJ4FM & DHFM

	Isorhamnetin 3-*O*-neohesperidoside	1.08E + 04	9.00E + 00	8.33E-04	1.01	DHFM
	Kaempferol 3-*O*-robinobioside	3.84E + 04	9.00E + 00	2.34E-04	1.10	DHFM
	Syringetin	6.33E + 04	9.00E + 00	1.42E-04	1.60	DHFM
**FLAVANONE**						
DHPM vs. XJ4PM	Hesperetin *O*-malonylhexoside	9.00E + 00	1.22E + 05	1.35E + 04	1.87	XJ4PM
	Phloretin	3.12E + 05	6.77E + 06	2.17E + 01	1.06	XJ4PM & DHPM
	Hesperetin 7-*O*-neohesperidoside (Neohesperidin)	1.39E + 07	1.87E + 05	1.35E-02	1.21	XJ4PM & DHPM
DHFM vs. XJ4FM	Naringenin	9.00E + 00	1.92E + 05	2.13E + 04	1.72	XJ4FM
	Hesperetin *O*-malonylhexoside	9.00E + 00	1.55E + 05	1.72E + 04	1.70	XJ4FM
	Naringenin 7-*O*-neohesperidoside (Naringin)	9.00E + 00	9.60E + 04	1.07E + 04	1.66	XJ4FM
	Hesperetin *O*-Glucuronic acid	9.00E + 00	4.25E + 04	4.72E + 03	1.58	XJ4FM
	Isosakuranetin-7-neohesperidoside (Poncirin)	1.02E + 03	1.16E + 04	1.14E + 01	1.11	XJ4FM & DHFM
**ISOFLAVONE**						
DHPM vs. XJ4PM	Genistein 7-*O*-Glucoside (Genistin)	9.00E + 00	1.87E + 05	2.08E + 04	1.92	XJ4PM
	Prunetin	1.03E + 03	3.49E + 04	3.40E + 01	1.31	XJ4PM & DHPM
DHFM vs. XJ4FM	Rotenone	9.00E + 00	4.27E + 04	4.74E + 03	1.58	XJ4FM
	Genistein 7-*O*-Glucoside	9.00E + 00	2.79E + 04	3.10E + 03	1.08	XJ4FM
	Glycitein	9.00E + 00	9.10E + 03	1.01E + 03	1.43	XJ4FM
	Calycosin	9.00E + 00	6.75E + 03	7.50E + 02	1.40	XJ4FM
**POLYPHENOL**						
DHPM vs. XJ4PM	Gallocatechin-gallocatechin	6.36E + 05	1.96E + 07	3.09E + 01	1.12	XJ4PM & DHPM
	(-)-Epiafzelechin	3.27E + 03	1.67E + 04	5.10E + 00	1.19	XJ4PM & DHPM
DHFM vs. XJ4FM	Gallocatechin-gallocatechin	9.00E + 00	2.90E + 07	3.23E + 06	2.11	XJ4FM
	(-)-Epiafzelechin	1.69E + 03	4.47E + 04	2.64E + 01	1.25	XJ4FM & DHFM
